# ﻿*Arundinellamainlingensis* (Poaceae), a new species from Xizang, China

**DOI:** 10.3897/phytokeys.257.151771

**Published:** 2025-06-03

**Authors:** Yu-Wei Tian, Xie-Yong Zhang, Ping Yan, Wen-Li Chen

**Affiliations:** 1 College of Life Sciences, Shihezi University, Shihezi, Xinjiang 832003, China Institute of Botany, Chinese Academy of Sciences Beijing China; 2 State Key Laboratory of Plant Diversity and Specialty Crops, Institute of Botany, Chinese Academy of Sciences, Beijing 100093, China Shihezi University Shihezi China; 3 University of Chinese Academy of Sciences, Beijing 100049, China University of Chinese Academy of Sciences Beijing China

**Keywords:** Morphology, phylogeny, Poaceae, taxonomy

## Abstract

*Arundinellamainlingensis* (Poaceae), a new species from Xizang, China is described and illustrated. This new species forms a sister group relationship with *A.yunnanensis* on the molecular phylogenetic tree, sharing common morphological characteristics such as ligule extremely short, glumes glabrous and 4−5 mm long and lemma with an apical awn. *Arundinellamainlingensis* differs from *A.yunnanensis* in having stems with 3 or 4 nodes, clustered basal leaves, densely covered with tubercle-based hairs on the margins and the adaxial surface, less cauline leaves and lower florets that are male or neuter with short anthers about 1.2 mm long. *Arundinellamainlingensis* is primarily found in south-eastern Xizang, including Nyingchi, Mainling and Bomê, whereas *A.yunnanensis* is only observed in Gongshan, Yunnan. These differences further reinforce the classification of the two as distinct species.

## ﻿Introduction

The tribe Arundinelleae Stapf represents an early-branching lineage within the supertribe Andropogonodae (Poaceae), comprising only two genera: *Arundinella* Raddi and *Garnotia* Brongn. (Soreng et al. 2022). The genus *Arundinella* encompasses approximately 50–55 species globally, predominantly located in tropical and subtropical regions of Asia (Clayton and Renvoize 1986; Soreng et al. 2022). In China, there are 21 species and two varieties, with the majority found in the south-western and southern regions (Sun and Phillips 2006). In Xizang, five species of *Arundinella* have been recorded, primarily distributed in the downstream areas of the Yarlung Tsangpo River in south-eastern Xizang, where the climate is warm and humid (Liou 1987). These species are valuable for soil conservation and provide quality forage for livestock (Sun 1990). *Arundinella* species are relatively easy to distinguish from closely-related genera by the terminal panicles, the pedicellate spikelets that are paired and disarticulating above the glumes and each spikelet with two florets. The lower floret is generally male or sterile, while the upper floret is bisexual, often awned and features a hairy callus at its base. In recent years, grass taxonomists have described five new taxa of *Arundinella*, based on specimens collected from China, Japan, India, Thailand and other regions. These include *A.thirunelliensis* Sunil, Ratheesh & Sivad. (Sunil et al. 2014), *A.pradeepiana* Sunil & Naveen Kum. (Sunil and Kumar 2014), A.nepalensisvar.xerophila Ibaragi (Ibaragi 2012), A.ripariasubsp.breviaristata Ibaragi (Ibaragi 2016) and *A.kokutensis* Teerawat. & Sungkaew (Teerawatananon et al. 2009). The identification of these new taxa has been supported by both morphology and molecular systematics.

*Arundinellayunnanensis* Keng was initially invalidly published as a naked name by Keng in 1957. In 1959, Keng cited the type specimen *C.W. Wang 65574*, collected from Gongshan, Yunnan, but did not provide a Latin description. Later, Sun and Hu (1980) supplemented this with a Latin diagnosis, thereby validating its publication. They also cited three specimens collected from Bomê and Mainling, Xizang (*Qinghai-Xizang Expedition 1460; Qinghai-Xizang Expedition 74-4650; Qinghai-Xizang Expedition 74-4648*). Since then, Chinese taxonomists have accepted the species concept of *A.yunnanensis* by Sun and Hu (Liou 1987; Sun 2003; Sun and Phillips 2006). Specimens from Nyingchi, Mainling and Bomê in Xizang have also been identified as *A.yunnanensis*.

Our study of specimens and field investigations revealed that samples, identified as *Arundinellayunnanensis* from south-eastern Xizang, although similar in certain morphological traits, such as extremely short ligule, glumes 4–5 mm long and glabrous and a lemma with an awn at the apex, exhibit clear morphological discontinuities in plant habit, hairiness of the leaves and anther length characteristics when compared to the type. We suspect that Sun and Hu (1980) may have misinterpreted *A.yunnanensis*, leading to the erroneous adoption of this species concept in major floras, such as *Flora of China*, *Flora Xizangica* and *Flora Yunnanica*. It is likely that the specimens from Xizang represent an undescribed new species. Based on field observations and herbarium specimen study, combined with phylogenetic analysis using nuclear genomic SNP data, we aim to resolve the taxonomic issues surround *A.yunnanensis*.

## ﻿Materials and methods

### ﻿DNA sampling extraction and sequencing

Five samples identified as *Aundinellayunnanensis* from different locations were selected: one from Gongshan, Yunnan (*C.W. Wang 65574*, NAS), cited by Keng (1959), the other four from Xizang, including one from Mainling (*Qinghai-Xizang Expedition 74-4648*, PE), supplemented by Sun and Hu (1980). Additionally, ten representative species within the genus were included. *Aundinella* contains four subgenera, of which three subgenera have been recorded in China and all have representative samples: two species from subgenus Chalynochlamis Franch., eight species from subgenus Arundinella and two species from subgenus Miliosaccharum Nees (Table 1). Three species are selected as outgroups and their sequences were obtained from the NCBI database: *Garnotiatenella* Janowski (accession: ERR13764505), *Coixlacryma*-*jobi* L. (accession: SRR9190398) and *Paspalumvaginatum* Sw. (accession: SRR24181711).

**Table 1.** Voucher Specimens for molecular sequencing (all specimens stored in PE, except for *C.W. Wang 65574* in NAS).

Subgenus (Keng, 1936)	Species	Collector & Collection number	Locality	Habitat	Elevation	This study
subg. Arundinella	*Arundinellayunnanensis* Keng	*C.W. Wang 65574*	Gongshan, Yunnan, China	hillside meadows	3000	*A.yunnanensis*
		*Qinghai-Xizang Expedition 74-4648*	Mainling, Xizang, China	hillside	3000	*A.mainlingensis* W.L. Chen & Y.W. Tian
		*W.L. Chen P5*	Nyingchi, Xizang, China	cliff	2895	
		*J.S. Ying, D.Y. Hong 650677*	Bomê, Xizang, China	cliff	2200	
		*Z.C. Ni, Y.C. Wang 1383*	Bomê, Xizang, China	forest	2700	
	*A.bengalensis* (Spreng.) Druce	*Z.Y. Cao 653*	Xueheng, Guizhou, China	hillside meadows	1400	
	*A.cochinchinensis* Keng	*Y.C. Liu, Y. Yang 434*	Yongde, Yunnan, China	s.n.	1056	
	*A.grandiflora* Hack.	*Northwest Yunnan Jinsha River Expedition 6526*	Heqing, Yunnan, China	forest	1700	
	*A.hookeri* Munro ex Keng	*X.W. Tian, Z.H. Zhang, Z.X. Tang 258*	Eryuan, Yunnan, China	wildness	2450	
	*A.nepalensis* Trin.	*Y.H. Shiu 12317*	Hong Kong, China	s.n.	s.n.	
	*A.pubescens* Merr. & Hack.	*s.n.* (PE00719778)	Shangcheng, Henan, China	s.n.	s.n.	
subg. Chalynochlamis	*A.anomala* Steud.	*Shandong University 199*	Qingdao, Shandong, China	s.n.	s.n.	
	*A.hirta* (Thunberg) Tanaka	*Z.Y. Liu 181446*	Chongqing, China	roadside	1200	
subg. Miliosaccharum	*A.setosa* Trin.	*Northwest Yunnan Jinsha River Expedition 4648*	Heqing, Yunnan, China	s.n.	2100	
	*A.Barbinodis* Keng ex B.S. Sun & Z.H. Hu	*Longxi Mountain Expedition 2341*	Jiangle, Fujian, China	forest edge	s.n.	

The total genomic DNA was extracted using the FastPure Plant DNA Isolation Mini Kit (DC104). Sequencing libraries were constructed with the MGIEasy Universal DNA Library Kit (BGI, Shenzhen, China). The sequencing was performed on the DNBSEQ-T7 high-throughput sequencing platform (Huazhong Agricultural University, Wuhan, China) using a paired-end (2 × 150 bp) sequencing strategy. The raw sequencing data were subjected to quality control using fastp v.0.23.2 (Chen et al. 2018). Reads with a length of less than 36 bp and an average base quality score of less than 20 were removed. Additionally, the first 15 bp of each read, which had poor sequencing quality, were trimmed.

### ﻿SNP calling and phylogenetic analysis

The clean reads were aligned to *Sorghumbicolor* (L.) Moench reference genome RTx436 (Wang et al. 2024a) using BWA-MEM v.0.7.17 (Li and Durbin 2009). The aligned reads were sorted using Samtools v.1.9 (Li et al. 2009). Biallelic single nucleotide polymorphisms (SNPs) were called using the HaplotypeCaller, CombineGVCFs and GenotypeGVCFs tools in GATK4 (McKenna et al. 2010). The SNPs were initially filtered using VariantFiltration with the default parameters: --filterExpression QD < 2.0 || FS > 60.0 || SOR > 3.0 || MQ < 40.0 || MQRankSum < -12.5 || ReadPosRankSum < -8.0. Further filtering was performed using VCFtools v.0.1.16 (Danecek et al. 2011) with the following parameters: --max-missing 0.7 --minQ 30 --min-meanDP 3. For phylogenetic analysis, the best-fit model selected by ModelFinder, based on the Bayesian Information Criterion, was TVM+F. A Maximum Likelihood tree was constructed using IQ-TREE v.1.6.12 (Nguyen et al. 2015) with 1000 ultrafast bootstrap replicates (-bb 1000).

Considering the potential bias that the reference genome may bring to phylogenetic analysis, we also employed a phylogenetic approach that does not rely on the reference genome. This method is based on k-mer statistics analysis using whole genome sequencing data and is suitable for constructing phylogenetic trees at the genus level (Wang et al. 2024b). KMC v.3.2.4 (REFRESH Bioinformatics Group 2024) and Mike v.1.0 (Wang et al. 2024b) were used to count k-mer (k = 21) from the whole genome sequencing reads and calculated the distance matrix and then R package ape (Paradis et al. 2004) was used to calculate the Neighbour-Joining tree. The trees were visualised and edited using FigTree v.1.4.4 (Rambaut 2018).

### ﻿Morphological research

In August and October 2021, field surveys and specimen collections were conducted in the Nyingchi, Xizang, where a total of 102 individuals from five populations were collected (Suppl. material 1: table S1). Additionally, 29 herbarium specimens from PE were examined, as well as 22 online specimen images from CDBI, FGC, HNWP, KUN, NAS, SZ and WUK (Suppl. material 1: table S2). Based on these over 150 specimens/individual, we made morphological observations and photographed key morphological characteristics.

## ﻿Results and discussion

**Figure 1.**
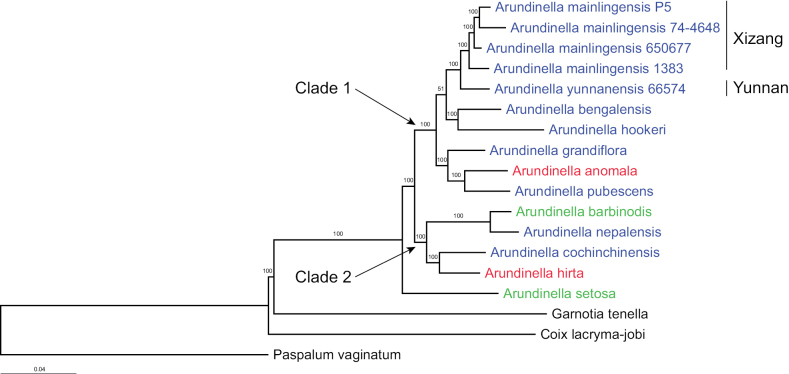
The Maximum Likelihood (ML) phylogenetic tree of *Arundinella* generated in IQ-TREE using nuclear genomic SNP data. Numbers above branches are ML ultrafast bootstrap values. The colour of the text indicates the subgenus to which the species belongs, with red representing subg. Chalynochlamis, green representing subg. Miliosaccharum and blue representing subg. Arundinella.

The Maximum Likelihood tree constructed using SNP data revealed that all samples collected from Xizang formed a monophyletic clade with 100% bootstrap support, sister to the sample from Gongshan, Yunnan (Fig. 1) and the Neighbour-Joining tree, based on the k-mer method, provided the same results (Suppl. material 2), suggesting that the samples collected from Xizang represent a distinct species.

**Figure 2.**
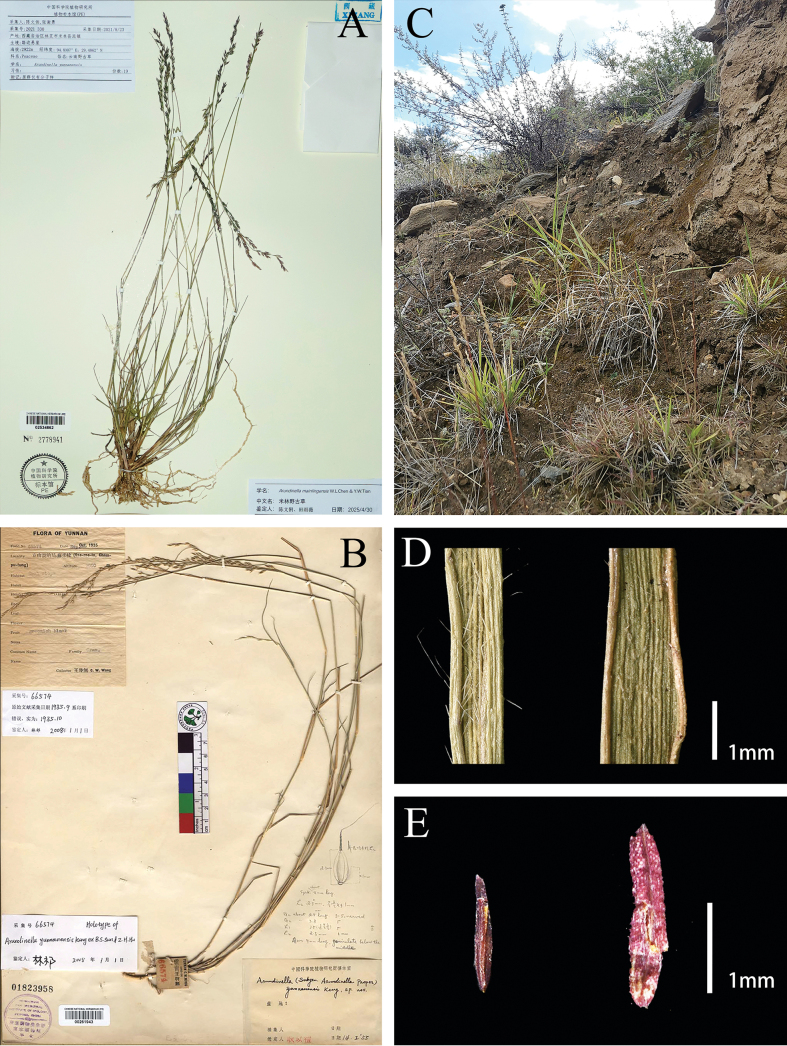
*Arundinellamainlingensis* and *A.yunnanensis*. **A** the holotype of *A.mainlingensis* (W.L. Chen, X.Y. Zhang 2021-330, PE, barcode 02534862) **B** the holotype of *A.yunnanensis* (C.W. Wang 66574, PE, barcode 00261943) **C** habitat of *A.mainlingensis***D** adaxial surface of basal leaves, left: *A.mainlingensis* densely covered with tubercle-based hairs on the margins, right: *A.yunnanensis* nearly glabrous **E** anthers, left: *A.mainlingensis*, right: *A.yunnanensis*.

We found that individuals from Xizang differ significantly from those from Gongshan, Yunnan, in terms of the culms with 3 or 4 nodes, clustered basal leaves densely covered with tubercle-based hairs on the margins and the adaxial surface, less cauline leaves, and anthers 1.2–1.5 mm long (Fig. 2, Table 2).

**Table 2.** A comparison between *Arundinellamainlingensis* and *A.yunnanensis*.

	*Arundinellamainlingensis*	*A.yunnanensis*
Culm height	15–45 cm	30–50 cm
Nodes	3–4	4–7
Basal leaves	clustered at the base	less basal leaves, not clustered
Leaf hairiness	margins and abaxial surface are densely covered with tubercle-based hairs	nearly glabrous
Lower floret	male or neuter	male
Anther length	1.2–1.5 mm	1.8–2 mm
Elevation	2200–4200 m	3000 m
Habitat	cliffs, under forests and forest meadows	hillside grasslands
Distribution	Xizang: Bomê, Mainling, Nyingchi	Yunnan: Gongshan

When Sun and Hu (1980) validated the publication of *Arundinellayunnanensis*, they misunderstood Keng’s (1957, 1959) definition of the species and incorrectly cited three specimens from Xizang (Qinghai-Xizang Expedition Team 1460, Qinghai-Xizang Expedition Team 74-4650 and Qinghai-Xizang Expedition Team 74-4648), leading to confusion in subsequent species identification. The former represents a new species – “*Arundinellamainlingensis* W.L. Chen & Y.W. Tian, sp. nov. ” – which is described and illustrated here. Additionally, the species boundaries of *A.yunnanensis* have been redefined. Currently, *A.yunnanensis* is only represented by four specimens, the type and three isotypes stored in PE and NAS (two specimens) and A, indicating that future field collections are needed.

**Figure 3.**
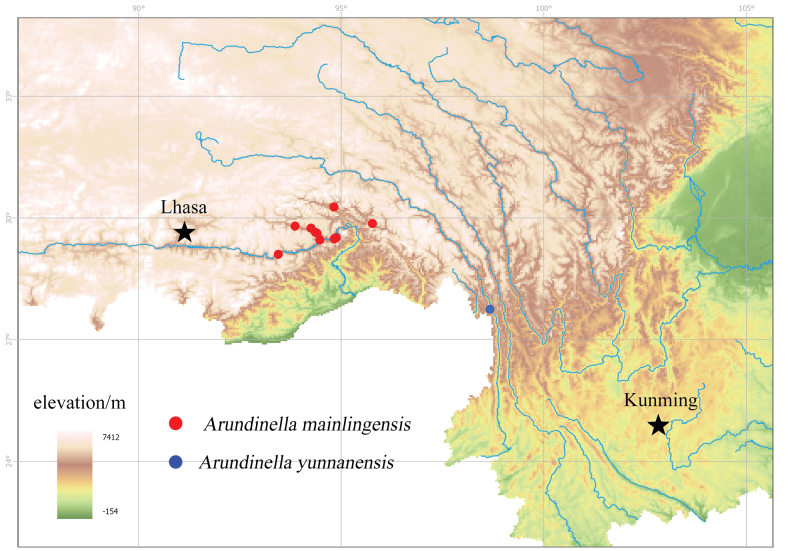
Distribution map of *Arundinellamainlingensis* and *A.yunnanensis*. Map source: https://www.tianditu.gov.cn/.

Liou (1980) divided the grass flora in Xizang into three regions and seven subregions. Amongst them, the South-east Tibetan forest-bush region has a warm and humid climate and possesses the richest diversity of Poaceae genera and species. This region is located on the northern slope of the Himalayas, the south-eastern part of the Gangdise and Nyenchen Tanglha Mountains and extends northeastwards, connecting with Qinghai, Sichuan and Yunnan. To the west, it borders the southern part of Ngari Area. The two sister species, *Aundinellamainlingensis* and *A.yunnanensis*, are distributed downstream of the Yarlung Tsangpo River in Xizang and the Nu River Basin in the western Hengduan Mountains of Yunnan, respectively (Fig. 3). Their distribution may reflect the close floristic connections between the south-eastern Xizang and the Hengduan Mountains regions.

Even though less than 25% of the species currently recognised in *Arundinella* were sampled, monophyly is supported with *A.setosa* forming the earliest diverging lineage (Fig. 1, Suppl. material 2). A previous molecular study using nrDNA ITS indicated an initial trichotomy split of three clades each containing 2–9 species (Jiang et al. 2022). The remaining species are divided into two major clades (Clade-1 and Clade-2) (Fig. 1). Keng (1936) placed the Asian species of *Arundinella* into four subgenera: *Psilachne* Keng, *Arundinella*, *Chalynochlamis* and *Miliosaccharum*, based on morphological characteristics, such as the presence or absence of awns on the lemma of the upper floret and the presence or absence of two lateral bristles, one on either side of the lemma, which was followed by Keng (1957) and Sun and Hu (1980). None of these subgenera appears monophyletic in our tree (Fig. 1). As Phipps (1967) argued that Keng’s subgenera appear somewhat arbitrary being based on so few characters, he then further divided 47 *Arundinella* species into 15 series. The monophyly of the four subgenera was also not supported in other recent molecular phylogenetic work (Jiang et al. 2022). The phylogenetic relationships amongst species within the genus *Arundinella* may need to be re-evaluated through in-depth studies, based on inclusion of plastid DNA and a larger sample of species within the genus.

### ﻿Taxonomic treatment

#### ﻿1. Arundinella mainlingensis

Taxon classificationPlantaePoalesPoaceae

W.L.Chen & Y.W.Tian sp. nov.

63FB5E31-E125-5BEF-A960-00A9E21F44D2

urn:lsid:ipni.org:names:77362643-1

Figs 3 , 4

##### Type.

China • Xizang, Mainling, roadside cliffs, 29°37′26.75″N, 94°24′13.68″E (DMS), 2922 m elev., 23 Aug 2021, *W.L. Chen, X.Y. Zhang 2021-330* (holotype: PE 02534862!; isotypes: PE 02534861! and 02534864!).

##### Description.

***Perennials***, usually densely tufted. ***Culms*** 15–35 cm tall, 1–1.5 mm in diam., glabrous, with 3 or 4 nodes. ***Leaf sheaths*** with long marginal hairs; ***ligules*** truncate, shorter than 0.1 mm; ***basal blades*** 3–7 cm long, 1–3 mm wide, firm, densely covered with tubercle-based hairs on the margins and adaxial surface, glabrous on the abaxial surface; basal leaves well-developed, clustered at the base, ***cauline blades*** 4–8 (–10) cm long, 1–3 mm wide, few. ***Panicles*** 7–15 cm long, contracted, rachis glabrous or slightly scabrid. ***Spikelets*** 4–5 mm long, 2-flowered, purple, glabrous, in pairs, one long-pedicelled, the pedicel 3 mm long and one short-pedicelled, the pedicel ca. 1 mm long; ***lower glumes*** 3–3.5 mm long, ovate-lanceolate, 3-veined; ***upper glumes*** 4–5 mm long, 5-veined; ***lower florets*** ca. 4 mm long, male or neuter; ***lemmas*** glabrous, 5-veined; ***upper florets*** ca. 2.5 mm long, bisexual; ***callus*** hairy, the hairs ca. 1/3 the length of the floret; ***lemmas*** of the upper floret slightly scabrid on back, 5-veined, apex awned, the awns ca. 3.5 mm long, geniculate with brown twisted column; ***paleas*** slightly longer than lemma, glabrous; ***anthers*** 1.2–1.5 mm long, purplish-red. ***Caryopses*** ca. 1.5 mm long, obovate-oblong, brownish.

**Figure 4.**
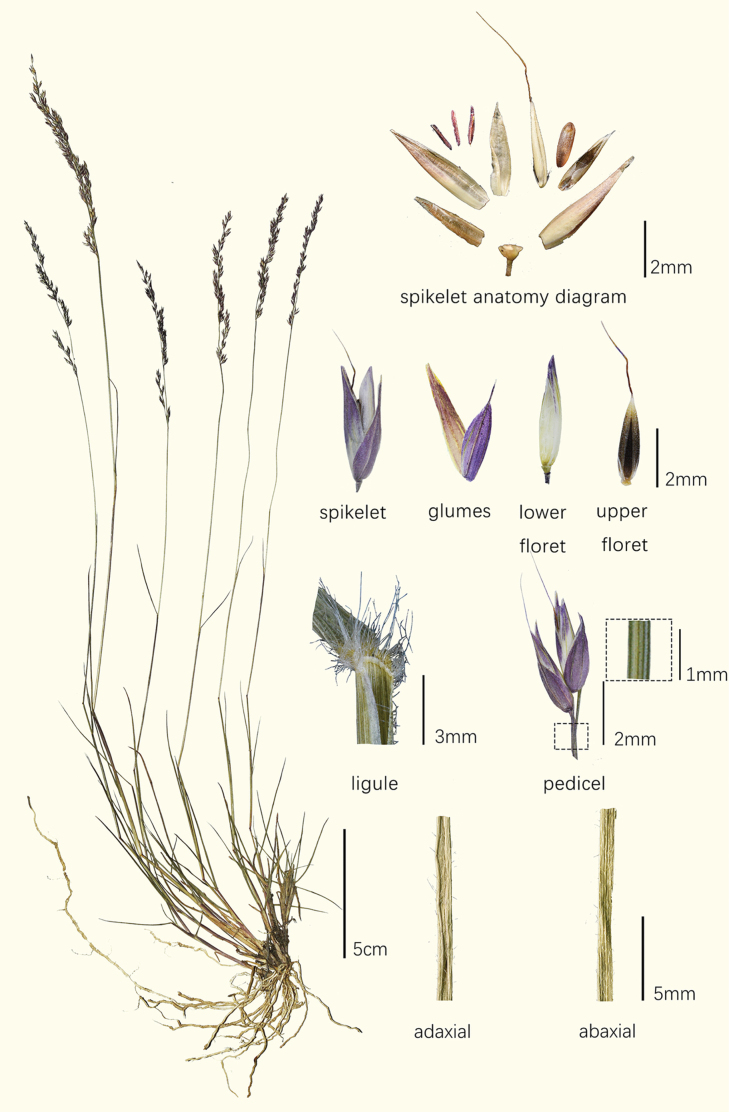
Holotype of *Arundinellamainlingensis* (*W.L. Chen, X.Y. Zhang 2021-330*).

##### Distribution and habitat.

The new species is known from Nyingchi, Mainling, Bomê in Xizang, China. It primarily grows on the cliffs along the middle reaches of the Yarlung Tsangpo River, as well as in forest margins and clearings at elevations from 2200–4200 m.

##### Phenology.

Flowering and Fruiting from July to September.

##### Etymology.

The specific epithet refers to the type locality, Mainling (Xizang, China).

##### Specimens examined.

**China. Xizang Bomê** • alpine meadow in spruce forest, 2700 m elev., 27 Aug 1980, *Z.C. Ni, Y.Z. Tian, Tsewang, Thubten 1383* (PE00261953, XZ0008588) • south hillsides, 3150 m elev., 3 Sep 1973, *J.Y. Zhang 1434* (PE01400609) • hillside meadows, 2200 m elev., 19 Jul 1965, *J.S. Ying, D.Y. Hong 650642* (PE00261949, 00261947, 00261959) • 3140 m elev., 20 Sep 1973, *Qinghai-Xizang Expedition 1460* (PE00261950, KUN320833); **Mainling** • cliffs and forest, 2895–2950 m elev., 26 Oct 2021, *W.L. Chen, Y.H. Ma IGDBP01-157* (PE) • 20 Sep 1974, *Qinghai-Xizang Expedition Vegetation Group 3295* (PE01469368, KUN320832) • hillsides, 3000 m elev., 12 Sep 1974, *Qinghai-Xizang Expedition 74-4650* (PE00261945) • hillsides, 2970 m elev., 17 Sep 1974, *Qinghai-Xizang Expedition 10700* (PE01803662, 01400610) • Under the shrubs, 3030 m elev., 12 Sep 2003, *X.F. Gao, W.G. Tu, Y.K. Qiao, H. He 7238* (CDBI0197670, 0197669) • forest, 3050 m elev., 11 Sep 2003, *X.F. Gao, W.G. Tu, Y.K. Qiao, H. He 7229* (CDBI0197671, 0197672) • forest, 3030 m elev., 12 Sep 2003, *X.F. Gao, W.G. Tu, Y.K. Qiao, H. He 7297* (CDBI0197673) • riversides, 3930 m elev., 1 Jul 1984, *B.C. Wu 84-456* (FGC0003249, 0003250, 0003254); **Nyingchi** • forest, 3050–3070 m elev., 26 Oct 2021, *W.L. Chen IGDBJ01-17* (PE) • forest, 3048 m elev., 24 Aug 2021, *W.L. Chen, X.Y. Zhang 2021-343* (PE) • roadside cliffs and forest, 2700 m elev., 12 Sep 1974, *Qinghai-Xizang Expedition 74-4648* (PE00261952) • forest, 2200 m elev., 9 Sep 1980, *Ecological Laboratory, Plateau Group 15438* (PE01400615, 01400614, 01400613) • forest, 2250 m elev., 11 Sep 1980, *Ecological Laboratory, Plateau Group 15484* (PE01400612) • forest, 3250 m elev., 16 Sep 1975, *Qinghai-Xizang Expedition 7966* (PE01400611) • meadows, 3100 m elev., *Ecological Laboratory, Plateau Group 15691* (PE01400608) • riversides, 3100 m elev., 16 Sep 1975, *Qinghai-Xizang Expedition 7649* (PE00261951, HNWP50590) • wastelands after deforestation, 3200 m elev., 16 Sep 1975, *Qinghai-Xizang Expedition 7875* (PE01469369) • hillsides, 2900 m elev., *s.n. 40* (PE01432412) • hillside meadows, 3040 m elev., 29 Jul 1965, *Y.T. Zhang, K.Y. Lang 1118* (PE00261958) • hillside meadows, 3100 m elev., 31 Jul 1965, *Y.T. Zhang, K.Y. Lang 1186* (PE00261957, 00261956) • hillside meadows, 3040 m elev., 28 Jul 1965, *Y.T. Zhang, K.Y. Lang 1065* (PE00261954) • 2100 m elev., 1 Jul 1984, *B.C. Wu 84-415* (FGC0003251, 0003252) • hillsides, 2950 m elev., 10 Jul 1984, *B.C. Wu 84-475* (FGC0003253, 0003255) • shrubs, 3005 m elev., 13 Aug 2018, *J. Hu, D.C. Wang, L. Li, L. Tang sc-a-084-B18* (CDBI0262139) • shrubs, 3005 m elev., 13 Aug 2018, *J. Hu, D.C. Wang, L. Li, L. Tang sc-a-084-B20* (CDBI0261963).

#### ﻿2. Arundinella yunnanensis

Taxon classificationPlantaePoalesPoaceae

Keng in B.S. Sun & Z.H. Hu, Acta Bot. Yunnanica 2(3): 326. 1980.

79AA4BD3-213D-5D52-925F-1104232B56FA

Arundinella yunnanensis Keng, Claves Gen. Sp. Gram. Prim. Sinic. 130. 1957, nom. inval.; Illus. Fl. China: Gramineae 730. fig. 678. 1959, nom. inval.

##### Type.

China • Yunnan, Gongshan, Suroula, hillside meadows, 3000 m elev., Oct 1935, *C.W. Wang 66574* (holotype: PE 00261943!; isotypes: A 00023142 [image!]; NAS 00566677[image!] and 00515176[image!]).

##### Description.

***Perennials***, loosely tufted. ***Culms*** 30–60 cm tall, 1–1.5 mm in diam., smooth and glabrous, with 4–7 nodes. ***Leaf sheaths*** glabrous; ***ligules*** shorter than 0.1 mm, nearly absent; ***basal blades*** 5–8 cm long, 1–3 mm wide, firm, flat or with involute margins, glabrous or occasionally with tubercle-based hairs; ***cauline blades*** 7–12 cm long, 2–4 mm wide. ***Panicles*** 10–15 cm long, contracted. ***Spikelets*** 4.5–5.2 mm long, 2-flowered, purple, glabrous, in pairs with one long-pedicelled, the pedicel 3 mm long and one short-pedicelled, the pedicel ca. 1 mm long; ***lower glumes*** ca. 3.5 mm long, 3-veined, ovate-lanceolate; ***upper glumes*** 4.2–5 mm long, 5-veined; ***lower florets*** ca. 4 mm long, staminate; ***lemmas*** 5-veined; ***upper florets*** ca. 3 mm long; ***lemmas*** of the upper floret slightly scabrid on back, 5-veined, apex awned, the awns ca. 3.5 mm long, geniculate with brown twisted column; ***paleas*** slightly longer than lemma; ***anthers*** 1.8–2 mm long, purplish-red. ***Caryopses*** ca. 1.6 mm long, obovate-oblong, brownish.

##### Distribution and habitat.

Found only in China in Gongshan, Yunnan, growing in hillside meadows at an elevation of 3000 m.

##### Phenology.

Flowering and Fruiting from July to September.

### ﻿Key to the species of *Arundinella* in Xizang and Yunnan

**Table:** 

1	Lemmas of upper floret awned	**1. *Arundinellahirta***
–	Lemmas of upper floret awnless	**2**
2	Awn of lemma without lateral bristles	**3**
–	Awn of lemma with two lateral bristles	**13**
3	Panicles densely spike-like	**4**
–	Panicles not spike-like	**5**
4	Panicles 10–30 cm long, plant usually over 1 m tall	**2. *A.bengalensis***
–	Panicles shorter than 10 cm, plant not exceeding 1 m	**3. *A.hookeri***
5	Culms and panicle branches glabrous	**6**
–	Culms and panicle branches hairy	**4. *A.nepalensis***
6	Panicle large, 20–70 cm long; plant usually over 1 m tall	**7**
–	Panicle small, not exceeding 20 cm long; plant not exceeding 1 m tall	**11**
7	Panicle usually lax, panicle axis exposed; leaves glabrous or with short hairs	**8**
–	Panicle dense, panicle axis often covered by branches; leaves usually with tubercle-based hairs	**10**
8	Spikelets 5 mm long; awn up to 4 cm, persistent	**5. *A.cochinchinensis***
–	Spikelets 3–4 mm, awn short and easily deciduous	**9**
9	Glumes setose along veins	**6. *A.tricholepis***
–	Glumes glabrous	**7. *A.decempedalis***
10	Spikelets 3–5 mm long, glumes unequal	**8. *A.nepalensis***
–	Spikelets 2–2.5 mm long, glumes subequal	**9. *A.parviflora***
11	Leaves 5–10 cm long, growing in alpine meadows at 3000 m elevation	**12**
–	Leaves 15–20 cm long, found only in riverbank rock crevices	**10. *A.rupestris***
12	Anthers ca. 2 mm long, basal leaves few, glabrous	**11. *A.yunnanensis***
–	Anthers ca. 1.2 mm long, basal leaves clustered, densely covered with tubercle-based hairs	**12. *A.mainlingensis***
13	Awn straight, culms herbaceous, nodes densely covered with white hairs	**13. *A.barbinodis***
–	Awn geniculate; culms blades firm, nodes with or without hairs	**14**
14	Spikelets 6–8 mm long; glumes densely covered with rigid tubercle-based hairs	**14. *A.khaseana***
–	Spikelets less than 6 mm long; glumes glabrous	**15**
15	Culms slender, 20–50 cm tall, spikelets 4.5–5 mm long	**15. *A.nodosa***
–	Culms robust, (35–) 60–180 cm tall, spikelets 5–6 mm long	**16. *A.setosa***

## Supplementary Material

XML Treatment for Arundinella mainlingensis

XML Treatment for Arundinella yunnanensis

## ﻿Additional information

### Author ORCIDs

Yu-Wei Tian https://orcid.org/0009-0002-6689-5903

Xie-Yong Zhang https://orcid.org/0000-0003-3785-4408

Wen-Li Chen https://orcid.org/0000-0002-5519-811X

### Data availability

All of the data that support the findings of this study are available in the main text or Supplementary Information. The whole-genome sequencing data for Arundinella species have been deposited in CNGB under accession CNP0007234.

## Supplementary materials

Supplementary material 1 Supplementary table data Data type docx Explanation note **table S1.** Field population observation and collection (all specimens are stored in PE); **table S2.** Additional specimen examined. https://binary.pensoft.net/file/1344116This dataset is made available under the Open Database License (http://opendatacommons.org/licenses/odbl/1.0/). The Open Database License (ODbL) is a license agreement intended to allow users to freely share, modify, and use this Dataset while maintaining this same freedom for others, provided that the original source and author(s) are credited.Yu-Wei Tian

Supplementary material 2 The Neighbour-Joining phylogenetic tree of *Arundinella* generated from k-mer analysis Data type svg Explanation note KMC v.3.2.4 and Mike v.1.0 were used to count k-mer (k = 21) from the whole genome sequencing reads and calculate the distance matrix, then R package ape was used to calculate the Neighbour-Joining tree. https://binary.pensoft.net/file/1344117This dataset is made available under the Open Database License (http://opendatacommons.org/licenses/odbl/1.0/). The Open Database License (ODbL) is a license agreement intended to allow users to freely share, modify, and use this Dataset while maintaining this same freedom for others, provided that the original source and author(s) are credited.Yu-Wei Tian, Xie-Yong Zhang, Ping Yan, Wen-Li Chen
