# Supplementary material for: ﻿Arundinellamainlingensis (Poaceae), a new species from Xizang, China
Source: PhytoKeys. 2025 Jun 3;257:65–78. doi: 10.3897/phytokeys.257.151771 (PMC12152490; doi:10.3897/phytokeys.257.151771)
Supplement: Supplementary material 1 — Supplementary table data [file phytokeys-257-065_article-151771__-s001.docx]

| **Table S1.** Field population observation and collection (all specimens are stored in PE) | | | | | | |  |  |
| --- | --- | --- | --- | --- | --- | --- | --- | --- |
| **Species** | | **Collector & Collection number** | **Locality** | **Collection time** | **Habitat** | **Elevation** | **Population Sampling Size** | |
| *Arundinella mainlingensis* | | W.L. Chen, Y.H. Ma IGDBP01-157 | Mainling, Xizang, China | 2021-10-26 | forest | 2895-2950m | 6 individuals | |
|  |  |  |  |  | cliff | 2895-2950m | 68 individuals | |
|  |  | W.L. Chen IGDBJ01-17 | Nyingchi, Xizang, China | 2021-10-25 | forest | 3050-3070m | 6 individuals | |
|  |  | W.L. Chen, X.Y. Zhang 2021-330 | Mainling, Xizang, China | 2021-9-16 | cliff | 2022m | 19 individuals | |
|  |  | W.L. Chen, X.Y. Zhang 2021-343 | Nyingchi, Xizang, China | 2021-9-16 | forest | 3048m | 3 individuals | |

| **Table S2.** Additional specimen examined | |  |
| --- | --- | --- |
| **Species** | **Additional Spedmens Examined** | |
| *Arundinella mainlingensis* | **CHINA. Xizang Bomê:** 2700 m, 27 Aug 1980, *Z.C. Ni, Y.Z. Tian, Tsewang, Thubten 1383* (PE00261953, XZ0008588); 3150m, 3 Sep 1973, *J.Y. Zhang 1434* (PE01400609); 2200m, 19 Jul 1965, *J.S. Ying, D.Y. Hong 650642* (PE00261949, 00261947, 00261959); 3140m, 20 Sep 1973, *Qinghai-Xizang Expedition 1460* (PE00261950, KUN320833).  **CHINA. Xizang Mainling:** 20 Sep 1974, *Qinghai-Xizang Expedition Vegetation Group 3295* (PE01469368, KUN320832); 3000m, 12 Sep 1974, *Qinghai-Xizang Expedition 74-4650* (PE00261945); 2970m, 17 Sep 1974, *Qinghai-Xizang Expedition 1070* (PE01803662, 01400610); 3030m, 12 Sep 2003, *X.F. Gao, W.G. Tu, Y.K. Qiao, H. He 7238* (CDBI0197670, 0197669); 3050m, 11 Sep 2003, *X.F. Gao, W.G. Tu, Y.K. Qiao, H. He 7229* (CDBI0197671, 0197672); 3030m, 12 Sep 2003, *X.F. Gao, W.G. Tu, Y.K. Qiao, H. He 7297* (CDBI0197673); 3930m, 1 Jul 1984, *B.C. Wu 84-456* (FGC0003249, 0003250, 0003254).  **CHINA. Xizang Nyingchi:** 2700 m, 12 Sep 1974, *Qinghai-Xizang Expedition 74-4648* (PE00261952); 2200m, 9 Sep 1980, *Ecological Laboratory, Plateau Group 15438* (PE01400615, 01400614, 01400613); 2250m, 11 Sep 1980, *Ecological Laboratory, Plateau Group 15484* (PE01400612); 3250m, 16 Sep 1975, *Qinghai-Xizang Expedition 7966* (PE01400611); 3100m, *Ecological Laboratory, Plateau Group 15691* (PE01400608); 3100m, 16 Sep 1975, *Qinghai-Xizang Expedition 7649* (PE00261951, HNWP50590); 3200m, 16 Sep 1975, *Qinghai-Xizang Expedition 7875* (PE01469369); 2900m, s.n. 40 (PE01432412); 3040m, 29 Jul 1965, *Y.T. Zhang, K.Y. Lang 1118* (PE00261958); 3100m, 31 Jul 1965, *Y.T. Zhang, K.Y. Lang 1186* (PE00261957, 00261956); 3040m, 28 Jul 1965, *Y.T. Zhang, K.Y. Lang 1065* (PE00261954); 2100m, 1 Jul 1984, *B.C. Wu 84-415* (FGC0003251, 0003252); 2950m, 10 Jul 1984, *B.C. Wu 84-475* (FGC0003253, 0003255); 3005m, 13 Aug 2018, *J. Hu, D.C. Wang, L. Li, L. Tang sc-a-084-B18* (CDBI0262139); 3005m, 13 Aug 2018, *J. Hu, D.C. Wang, L. Li, L. Tang sc-a-084-B20* (CDBI0261963). | |
